# Preparation of high-permeance ceramic microfiltration membranes using a pore-sealing method

**DOI:** 10.1039/c9ra09805d

**Published:** 2020-02-04

**Authors:** Wu Qin, Yi Zhang, Jianqing Wu

**Affiliations:** School of Materials Science and Engineering, South China University of Technology Guangzhou 510640 China imjqwu@scut.edu.cn +86-20-87110273 +86-20-87111669; School of Materials Science and Energy Engineering, Foshan University Foshan 528000 China

## Abstract

A pore-sealing method for preparation of high-permeance alumina microfiltration (MF) membranes free of any intermediate layers is presented. It involves sequential coating of a polyvinyl butyral (PVB) layer and an alumina membrane precursor on the surface of the macroporous alumina support. An alumina MF membrane with no intermediate layers can be obtained on the support after pyrolysis of the PVB interlayer. The interlayer-free membrane prepared by this method has an average pore diameter of 0.26 μm and a water permeance of 1468 ± 81 L m^−2^ h^−1^ bar^−1^ which is prominently higher than that of the ceramic membranes prepared with other techniques. The conspicuous increase of water permeance is speculated mainly due to the filtration resistance decrease of the interlayer-free ceramic membrane.

## Abstract 2

A pore-sealing method for preparation of high-permeance alumina microfiltration (MF) membranes free of any intermediate layers is presented.

## Graphical abstract

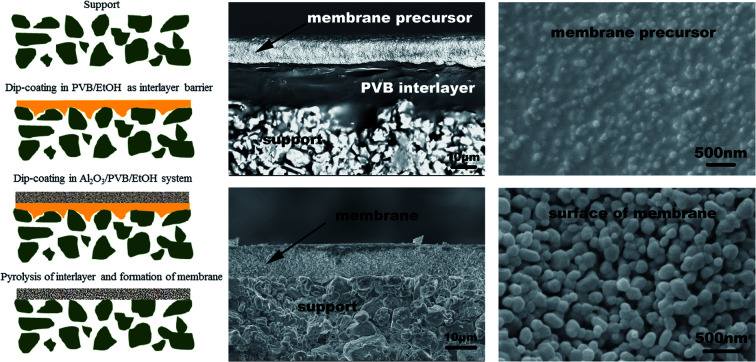

## 1. Introduction

Ceramic membranes with all sorts of insoluble metal oxides such as Al_2_O_3_, ZrO_2_, SiO_2_ and TiO_2_ (ref. 1) have shown interesting separation and processing properties. Usually, a porous ceramic membrane is characterized by a multi-layered asymmetric structure which is composed of a thicker (1–5 mm) support layer with relatively large pores (1–15 μm) to provide mechanical integrity for the membrane systems, an intermediate layer to reduce pore size to mesoporous dimensions and a much thinner (10–50 μm) top layer with small (2–500 nm) and selective pores for selective separation.^2,3^ Typically, the fabrication of a porous membrane involves multiple steps where the coating of intermediate layers and the final separation layer is normally performed on the prepared support layer. Multiple high-temperature sintering processes are normally required to combine these layers. In fact, the complexity and the expensive starting materials during membrane fabrication have concertedly resulted in the high production cost of ceramic membranes.^4–6^ Membrane-forming particles of the fine separation layer are easy to penetrate into the large pores of the support layer under capillary force, which results in pore blockings. In this way, the intermediate layers which bridge the pore size differences between the support layer and the top separation layer seem necessary.^7^ However, the intermediate layers lead to the rapid increase of membrane filtration resistance accompanied with the sharp decrease of water permeance.

In the literatures, Bayat *et al.*^8^ reported that a γ-Al_2_O_3_ multilayer ultrafiltration (UF) membrane on an α-alumina (α-Al_2_O_3_) substrate was successfully fabricated *via* the sol–gel processing method. The optimum permeate flux of the membrane was indentified as 112.7 L m^−2^ h^−1^ bar^−1^. Zou *et al.*^9^ designed one-step preparation of high-performance bi-layer α-alumina ultrafiltration membranes supported on coarse tubular substrates by co-sintering process. In this approach, boehmite sol and alumina nanoparticles were mixed in different ratios for the fabrication of MF layer and the UF layer. The membrane thickness of the MF layer and the UF layer was controlled to be 40–50 μm and about 1 μm, respectively. Recently, a method called “precursor film firing method” is proposed to improve the permeance of ceramic MF membranes by avoiding intermediate layers and dip-coating process, and efficiently control the thickness of the separation layer.^10^ Moreover, a sacrificial interlayer-based technique has been used to produce membranes without any intermediate layers.^7^

Some different polymers such as polyvinyl alcohol (PVA), polyvinyl pyrrolidone (PVP) and polyvinyl butyral (PVB) can be used as sacrificial phases.^11–13^ The use of these polymer binders can improve the drying of the wet film and avoid cracks in the membrane precursor. In this study, we design a pore-sealing method for preparation of high-permeance alumina MF membrane free of any intermediate layers. This method involves sequential coatings of a PVB layer and an alumina membrane precursor on the surface of the macroporous alumina support. In this approach, a high-permeance alumina MF membrane with no intermediate layers can be prepared after pyrolysis of the PVB interlayer.

## 2. Material and methods

**Fig. 1:**
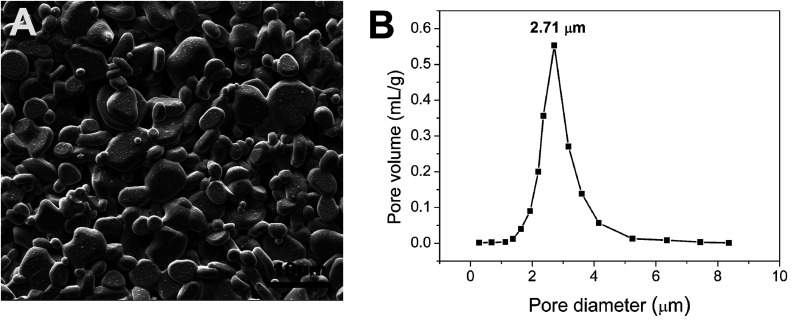
SEM surface morphology and pore size distribution of the pre-treated support.

The commercial tubular α-Al_2_O_3_ supports provided by Foshan Ceramics Research Institute Co., Ltd (Guangdong, China) were immersed in 5% HCl solution for 30 min, followed by heat treatment at 650 °C for 60 min. The above pretreated supports were characterized by an open porosity of (48.6 ± 3.2)% and an average pore size of 2.71 μm (Fig. 1), along with a water permeance of 8625 ± 172 L m^−2^ h^−1^ bar^−1^. The pretreated supports were soaked in the 5 wt% PVB/EtOH solution^14^ by ultrasound for 30 min until no air bubbles were visible and the supports were completely filled with the PVB/EtOH solution, and then pulled out slowly. After volatilization of ethanol in the wet film a PVB film formed on the support surface. The surface pores of the support were sealed compactly with the PVB film. Both ends of the support were sealed with masking tape, and dip coating was performed on the support with the membrane-forming suspensions which composed of 5 wt% PVB (15–35 mPa s, butyl aldehyde: 45–49%, Sinopharm Chemical Reagent Co., Ltd, China), 80 wt% ethanol absolute (AR, Tianjin Yongda Chemical Reagent Co., Ltd, China) and 15 wt% α-Al_2_O_3_ (99.9% purity, *d*_50_ = 100 nm, Taimei Chemicals Co., Ltd Nagano-ken, Japan), and the support was immediately pulled out at the speed of 5 cm s^−1^.^15^ Based on our previous work,^15^ the membrane precursor was dried slowly at ambient temperature and finally sintered at 1300 °C for 2 h at the heating rate of 5 °C min^−1^, which was accompanied with natural cooling.

The porosities of the supports were measured according to the Archimedes method and the theoretical density of α-Al_2_O_3_ was taken as 3.99 g cm^−3^. Pore size of the support and membrane was determined by the gas bubble pressure method based on the ASTM Publication F316-03(2011). The support and membrane was saturated with deionized water (18 MΩ cm) before the pressure was applied so as to avoid non-stationary transient effects. Morphologies of the support and membrane were observed by scanning electron microscope (ZEISS EVO 18, Germany). A Fully Automated Fluid and Gas Handling System (OSMO Inspector 2.0, Convergence, Netherlands) was utilized to measure the water permeance of the support and membrane.

## 3. Results and discussion

**Fig. 2:**
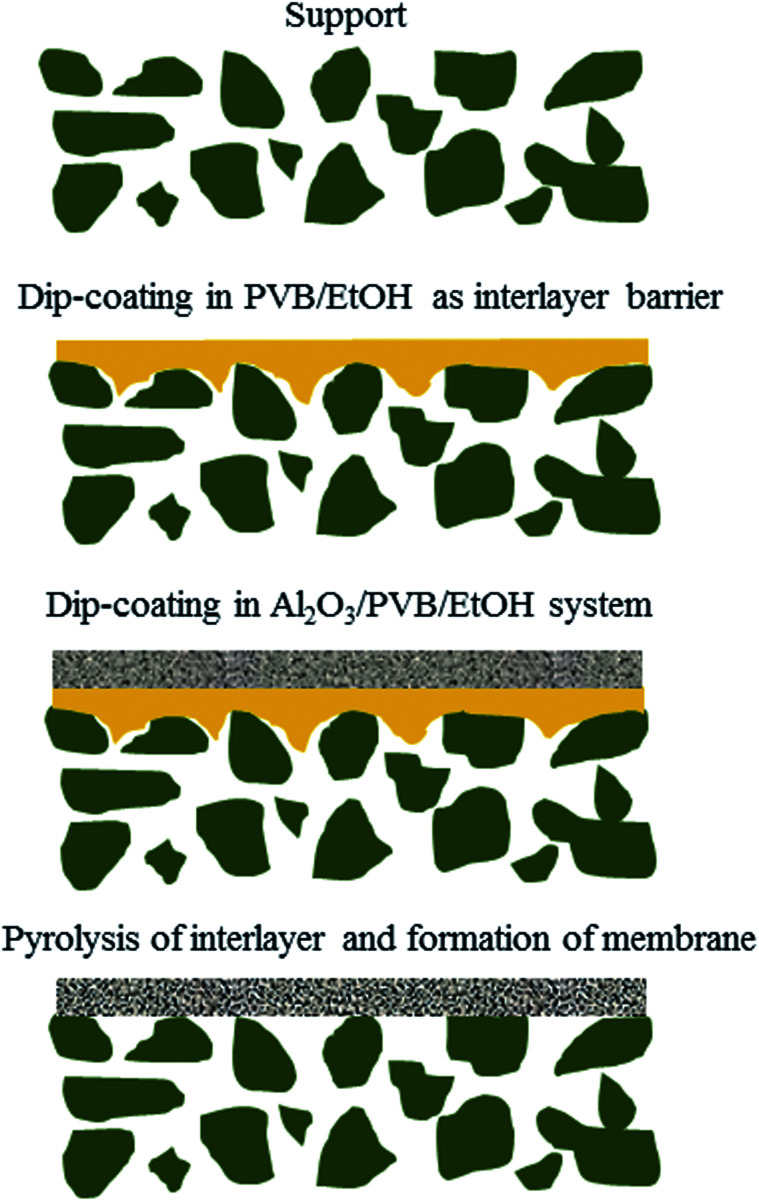
Schematic illustration of a pore-sealing method for preparation of high-permeance ceramic microfiltration membrane.

**Fig. 3:**
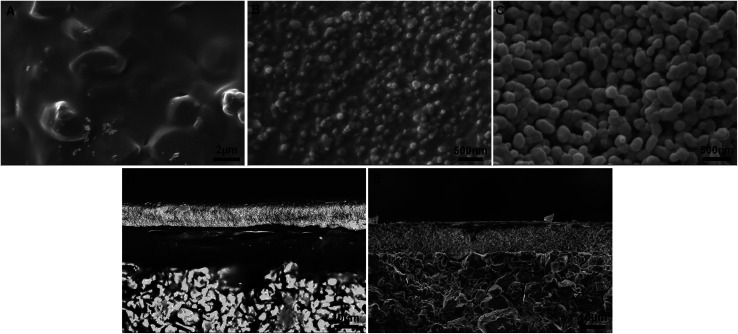
SEM morphologies of the PVB interlayer, membrane precursor and sintered membrane: (A) surface of the PVB interlayer; (B) surface of the alumina membrane precursor; (C) surface of the sintered membrane; (D) cross-section of the three-tier structure; (E) cross-section of the sintered membrane.

A schematic diagram of a pore-sealing method for preparation of high-permeance ceramic microfiltration membrane is shown in Fig. 2. Ultrasonic immersion of the support in 5 wt% PVB/EtOH solution aims at making a defect-free PVB film to seal the surface pores of the support completely. The PVB molecule has both butyral (hydrophobic) group and hydroxyl (hydrophilic) group. It is as easy as a pie for the PVB/EtOH solution to saturate the support. The cured PVB film is embedded in the surface pores of the support. The intrinsic viscosity and hydrophilicity of the PVB molecules facilitate the tight adhesion of a smooth PVB film on the hydrophilic surface of the support (Fig. 3A). After dip coating in the Al_2_O_3_/PVB/EtOH suspensions, an alumina membrane precursor is formed on the PVB film. The expanded conformation of the PVB molecules in the solvents is effective for dispersion of the fine alumina particles.^16^ All the gaps among the membrane-forming particles are completely filled with the cured PVB (Fig. 3B). The crack-free membrane precursor combines compactly with the PVB interlayer due to the amphipathic property of PVB (Fig. 3D). For the as-deposited layers, the thickness of the PVB interlayer and the membrane precursor is about 18 μm and 13 μm, respectively. The three-tier structure shows that a PVB interlayer bridges the membrane precursor and the support, and stops the fine alumina particles from penetrating into the pores of the support. A defect-free alumina membrane combines tightly with the support (Fig. 3E) after the burning-off of the PVB interlayer at 1300 °C for 2 h. The thickness of the sintered membrane is around 12 μm. During the continuous melting and pyrolysis of the PVB interlayer that acts as a pore former, the top membrane precursor is facilitated to conglutinate the support surface and a great deal of pores are formed evenly in the prepared membrane (Fig. 3C).

**Fig. 4:**
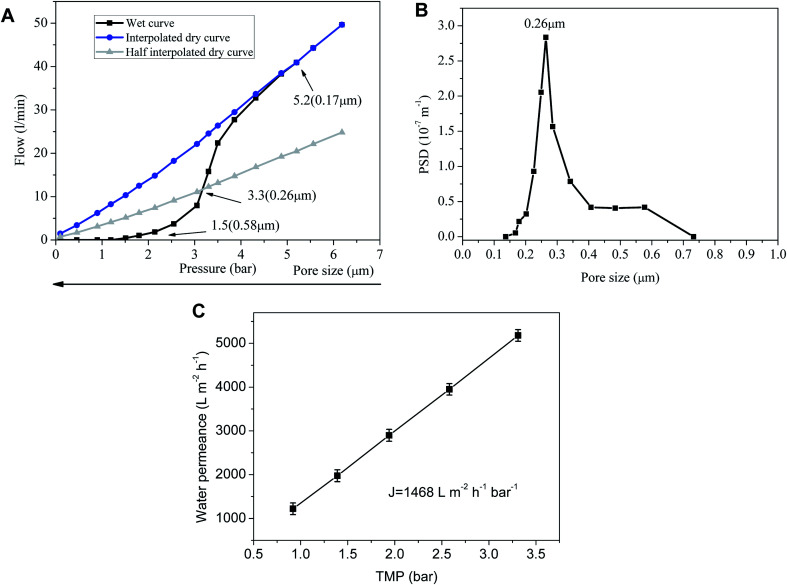
Pore size distribution (A and B) and water permeance (C) of the sintered membrane.

The pore size of the sintered membrane is measured at 25 °C according to ASTM Publication F316-03(2011). When the transmembrane pressure difference reaches 1.5 bar, the first bubble point is detected, correspsonding to the largest pore size of 0.58 μm (Fig. 4A). Nonlinear growth of the gas flow is observed with the increase of transmembrane pressure difference, which indicates more and more wet pores are opened. Sharp increase of the gas flow at the transmembrane pressure difference of 3.3 bar signifies that the pore size of the membrane is centered at 0.26 μm. The gas flow of wet pores finally turns to increase linearly with transmembrane pressure difference after the rest of the wet pores are completely opened, which accords with the Hagen–Poiseuille equation. Correspondingly, the membrane is endowed with a water permeance of 1468 ± 81 L m^−2^ h^−1^ bar^−1^ (Fig. 4C) which is remarkably higher than that of the inorganic membranes prepared by other techniques^17–22^ (Table 1). The possible reasons for the high water permeance are that the PVB interlayer restrains the fine membrane-forming particles from infiltrating into the support and blocking the pores, and that the absence of intermediate layers leads to the sharp decrease of membrane filtration resistance. But for intermediate layers, membrane-forming particles with very small size are very easy to infiltrate into the pores of the membrane support under capillary force during dip coating. The pore-blockings of the support makes the filtering performance decrease sharply. Although intermediate layers can bridge the pore size differences between the support layer and the top separation layer and apparently reduce the infiltration of membrane particles into the support, they will increase significantly the thickness of the effective separation layers. The thicker the effective separation layers are, the higher the filtration resistance will be. In this way, the filtration resistance of the membrane will increase dramatically with the augment of the separation layers thickness.^23,24^

**Table 1:** Comparisons water permeance of the alumina membrane prepared in this work with those in other literatures

Membrane	Average pore size (μm)	Water permeance (L m^−2^ h^−1^ bar^−1^)	Thickness (μm)	Reference
α-Al_2_O_3_	0.26	1.468 × 10^3^	∼12	This work
ZrO_2_	—	1 × 10^3^	3–4	17
α-Al_2_O_3_	1.25	799	—	18
TiO_2_	0.1	740	15–20	19
α-Al_2_O_3_	0.76	1.93 × 10^3^	—	20
Clay	0.18	867	—	21
Carbon	1.0	450.7	—	22

**Fig. 5:**
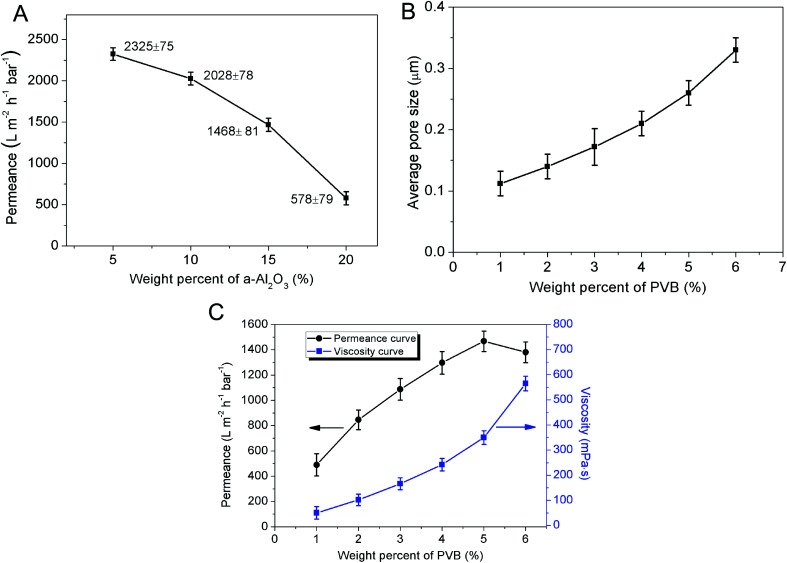
(A) The relation between permeance and the weight percent of α-Al_2_O_3_ in the suspension; (B) average pore size of the membrane based on the weight percent of PVB in the suspension; (C) permeance of the membrane and viscosity of the suspension with the variation of PVB content.

The weight percentage of α-Al_2_O_3_ in the membrane forming suspension has an influence on the permeance of membrane. In the range from 5 wt% to 20 wt%, the permeance of membrane degrades from 2325 ± 75 L m^−2^ h^−1^ bar^−1^ to 578 ± 79 L m^−2^ h^−1^ bar^−1^ and the reduction rate accelerates with the increase of α-Al_2_O_3_ content (Fig. 5A). This is probably because increment of solid content of the suspension would cause the increase of membrane thickness and pore length, which can greatly result in the enlargement of membrane resistance. The effect of PVB content on the permeance of membrane cannot be neglected yet. Fig. 5C shows when the weight percent of PVB was 1%, the permeance of membrane is as low as 489 ± 88 L m^−2^ h^−1^ bar^−1^. One function of the PVB is to increase the viscosity of the suspension. The viscosity of the suspension with 1% PVB is so low that the particles in the suspension would easily infiltrate into the support under capillary force. And this results in plugging part of the pores in the support and increasing the resistance. With the augment of PVB content the viscosity of the suspension increases. Due to the formation of giant network structure in the suspension, the particles are impeded to infiltrate into the support. Meanwhile, PVB as a pore former can also increase the porosity and average pore size of the membrane (Fig. 5B). However, it doesn't mean the higher content of PVB, the better permeation performance of the membrane. As Fig. 5C shows, the increase rate of permeance gradually slows down with increasing PVB content whereas the viscosity of suspension is enhanced. The reason is may be that an excess of PVB may simultaneously increase the viscosity of the suspension and further enlarge the thickness of membrane. When PVB content is above 5 wt%, there is a turning point in the curve of permeance *vs.* PVB content, and permeance of the membrane descend due to the sharp ascent of resistance. This can be explained that the effects of pore length increment and pore connectivity decline surpass the effect of porosity and pore size increase. Control of PVB content is substantially effective to adjust the suspension viscosity and further regulate thickness of the precursor membrane.

## 4. Conclusions

A high-permeance alumina microfiltration membrane free of any intermediate layers has been prepared by a pore-sealing method. The tubular support for membrane is firstly covered with a PVB film as a barrier that seals the surface pores completely then dip-coated in the Al_2_O_3_/PVB/EtOH suspensions, which is followed by calcination at 1300 °C for 2 h and natural cooling. The PVB interlayer prevents the fine membrane-forming particles from infiltrating into the support and blocking the pores. The absence of intermediate layers facilitates the membrane resistance to decrease sharply. These two reasons may account for the high water permeance of the membrane. The interlayer-free alumina membrane prepared by this pore-sealing method has an average pore size of 0.26 μm and a water permeance of 1468 ± 81 L m^−2^ h^−1^ bar^−1^ which is remarkably higher than that of the inorganic membranes prepared by other techniques.

## Conflicts of interest

There are no conflicts to declare.

## Supplementary Material
